# Histopathological and Immunohistochemical Characterization of Sebaceous Adenoma and Epithelioma in Dogs

**DOI:** 10.3390/ani14101457

**Published:** 2024-05-14

**Authors:** Sanggu Kim, Preeti Kumari Chaudhary, Sachin Upadhayaya, Kwang Won Seo, Soochong Kim

**Affiliations:** College of Veterinary Medicine, Chungbuk National University, Cheongju 28644, Republic of Korea; tkdrnfld@naver.com (S.K.); chaudharypreety11@gmail.com (P.K.C.); dr.supadhayaya@gmail.com (S.U.); vetskw16@cbu.ac.kr (K.W.S.)

**Keywords:** sebaceous gland, adenoma, epithelioma, histopathology, immunohistochemistry, dogs

## Abstract

**Simple Summary:**

Sebaceous gland tumors are less studied than other tumors because they have a relatively good prognosis and low incidence compared to other tumors. In particular, sebaceous gland tumors have received little attention in veterinary medicine, leading to diagnostic uncertainty. However, unlike sebaceous adenoma, epithelioma can metastasize and has a high mortality rate thus accurate diagnosis is essential. The present study aimed to validate and discuss the diagnostic criteria for sebaceous adenoma and epithelioma in dogs.

**Abstract:**

Sebaceous gland tumors are neoplasms originating from the sebaceous gland and are the third most common type of skin tumor, accounting for 21–35% of all cutaneous neoplasms in dogs. According to their histopathological characteristics, sebaceous gland tumors can be classified into adenoma as a benign tumor and epithelioma as a malignant tumor. Sebaceous epithelioma is distinguished from sebaceous adenoma by containing 90% or more reserve cells. However, this simple numerical criterion is insufficient to histologically distinguish between epitheliomas and adenomas. In addition, sebaceoma in humans, a similar tumor to sebaceous epithelioma, is a term used for tumors with more than 50% of reserve cells, unlike epithelioma. Therefore, we aimed to compare and characterize the histological and immunohistochemical profiles of comprehensive sebaceous adenoma, epithelioma, and borderline tumors that have more than 50% but less than 90% of reserve cells. A total of 14 canine sebaceous tumors were diagnosed as seven adenomas, four borderline tumors, and three epitheliomas. Histologically, the sebaceous adenomas showed nodules consisting of mature sebocytes surrounded by monolayer basaloid cells. In contrast, the portion of the reserve cells was increased, the portion of lipidized cells was decreased, and the majority of lipidized cells were found to be immature in sebaceous epithelioma. In the sebaceous adenomas, necrosis was not observed and mitotic figures were rarely seen. However, necrosis and mitotic figures were highly frequent in both borderline tumor and sebaceous epithelioma. Immunohistochemistry revealed that borderline tumor and sebaceous epithelioma showed significantly higher expression against Ki-67 than sebaceous adenoma. We conclude that it is more accurate to employ the cut-off value of 50% reserve cells in humans rather than the current 90% reserve cells for classifying sebaceous gland tumors in dogs, thereby providing new insight into the characterization of the sebaceous gland tumors.

## 1. Introduction

Sebaceous gland tumors represent the third most common type of skin tumor, accounting for 21–35% of all cutaneous epithelial tumors in dogs [1]. It can develop anywhere on the skin, but the most common areas are the head, abdomen, eyelid, and thorax [2]. It is also known to be more common in females (approximately 60%) and to occur at an average age of 9.5–10.5 years in dogs [3]. In addition, it has been reported to be most frequently found in Labrador retrievers (35%), followed by Pomeranians (13%) [4]. Although the etiology of the sebaceous gland tumors is not fully defined, it is known that a malignant variant of sebaceous gland tumors tends to occur when exposed to ultraviolet radiation, immunosuppression, and abnormal expression of hormonal receptors including the estrogen α receptor and the progesterone receptor [5,6].

Sebaceous gland tumors in dogs are sub-classified according to their histology as sebaceous adenoma, sebaceous epithelioma, and sebaceous carcinoma [7]. Sebaceous adenomas (benign) are well-circumscribed nodules composed of multiple large lobules of sebaceous cells showing normal maturation from peripheral basal cells, also called reserve cells, to large and pale lipid-laden central cells [8]. Sebaceous carcinomas (malignant) are poorly circumscribed nodules composed of irregular trabeculae of pleomorphic and atypical polygonal cells with variable degrees of cytoplasmic lipidization. These tumors typically lack significant numbers of reserve cells [8]. Sebaceous epitheliomas (low malignancy) are predominantly composed of reserve cells, with small numbers of interspersed intermediate and mature sebocytes [8].

Sebaceous epithelioma and adenoma are particularly important tumors because of their high incidence rates of 37% and 60% among sebaceous gland tumors, respectively [1]. In general, it is known that sebaceous adenoma is completely cured through excision therapy. However, since malignant tumors of the sebaceous gland show a 22% mortality rate, a 50% metastasize rate, and a 30–40% recurrence rate within 5 years in human, their accurate diagnosis is essential [5,9]. Similarly, in dogs, sebaceous epithelioma has a local recurrence rate of around 6%, and there have been reports of metastasis to the central nervous system (CNS) and lungs; thus, it is important to distinguish epithelioma from adenoma [10,11]. Nevertheless, studies related to histopathologic diagnosis of sebaceous gland tumors are lacking.

Sebaceous epithelioma, which is known to be malignant, has a criterion that distinguishes it from sebaceous adenoma. It has been shown that epithelioma is classified by containing 90% or more reserve cells in dogs [7]. However, this simple numerical criterion is insufficient to distinguish between sebaceous epithelioma and adenoma. In addition, unlike dogs, there is a sebaceoma in humans, similar to canine sebaceous epithelioma, which contains more than 50% of reserve cells [12]. Although human sebaceoma and canine sebaceous epithelioma have the predominance of basal cells over mature sebaceous cells in histology, different criteria used in the diagnosis of these tumors challenge the comparison and understanding of human and canine sebaceous tumors.

In the present study, we aimed to compare and characterize the histological and immunohistological profiles of comprehensive sebaceous adenomas, epitheliomas, and borderline tumors that have more than 50% but less than 90% of reserve cells. In order to characterize histological characteristics, we checked common histological features in malignant tumors including inflammation, necrosis, and mitotic figures. In addition, we performed immunohistochemistry (IHC) against Ki-67, which has been used as a proliferation marker for numerous tumors including canine sebaceous tumors, to objectively compare tumor aggressiveness [3,13,14]. 

## 2. Materials and Methods

### 2.1. Sample Collection

Biopsy samples of 14 dogs for the last 4 years (2019~2022) were provided to the Laboratory of Veterinary Pathology, Chungbuk National University, South Korea. Samples were surgically excised from various skin areas, including shoulders, ears, tail, and limbs.

### 2.2. Sample Processing and Hematoxylin and Eosin (H&E) Staining

All samples were fixed in 10% neutral buffered formalin (Samchun, Pyeongtaek, Republic of Korea), routinely trimmed, and processed. The processed samples were embedded within paraffin (Leica Biosystems, Richmond, IL, USA) and sectioned at a thickness of 5 μm to make tissue slides (Muto pure chemicals, Tokyo, Japan). The sections were deparaffinized with xylene (GD CHEM, Eumseong, Republic of Korea) and rehydrated with ethanol (GD CHEM, Eumseong, Republic of Korea). Sections were stained with H&E (Mirax, Mckinney, TX, USA). The H&E-stained sections were dehydrated with ethanol, cleared with xylene, and mounted with a coverslip (Deckglaser, Sondheim, Germany). The H&E-stained sections were scanned using a slide scanner (Olympus vs. 200, Tokyo, Japan) and histopathologically evaluated by histopathologists.

### 2.3. Immunohistochemistry (IHC)

IHC was conducted using antibodies against rabbit polyclonal Ki-67 (ab15580; Abcam, Waltham, MA, USA), previously used in canine tissues [15,16]. After deparaffinizing and rehydration with xylene and alcohol, slides were washed under tap water for 10 min. Antigen retrieval was performed by boiling washed slides in sodium citrate buffer (pH 6.0) (Biopure, Seoul, Republic of Korea) for 10 min in a microwave and kept at room temperature (RT) for 30 min. Then, antigen-retrieved slides were washed and incubated with 3% H_2_O_2_ (Samchun, Pyeongtaek, Republic of Korea) for 10 min at RT. The washed slides were blocked with 5% goat serum (Vectastain; Vector Laboratories, Newark, CA, USA) in phosphate-buffered saline (PBS) (Welgene, Gyeongsan, Republic of Korea) for 1 h, followed by washing with PBS. The slides were then incubated with primary antibodies at 4 °C overnight. After washing the slides with PBS, they were incubated with diluted biotinylated goat anti-rabbit secondary antibodies (Vectastain; Vector Laboratories, Newark, CA, USA) for 30 min at RT. Slides were washed in PBS and incubated with ABC reagent (Vectastain; Vector Laboratories, Newark, CA, USA) for 30 min at RT. After washing the slides again with PBS for 5 min, visualization was performed by incubating them in 3,3′-diaminobenzidine tetrahydrochloride solution (Vectastain; Vector Laboratories, Newark, CA, USA) for 10 min. The slides were washed and counterstained with hematoxylin for 1 min.

### 2.4. Histological Analysis

Histologically, 90% reserve cells, which is the cut-off value for distinguishing between sebaceous adenoma and epithelioma in canine, and 50% reserve cells, which is the cut-off value for distinguishing between sebaceous adenoma and sebaceoma in humans, were used to diagnose as follows: less than 50% reserve cells suggests sebaceous adenoma, more than 50% yet less than 90% suggests borderline tumor, and more than 90% suggests sebaceous epithelioma. The growth pattern of sebaceous adenoma was classified as exophytic and endophytic. In borderline tumor and sebaceous epithelioma, the growth pattern was classified as lobulated, papillary, and trabecular. Additionally, inflammation (inflammation if inflammatory cells are visible in more than 5% of the tumor area; no inflammation if inflammatory cells are visible in less than 5% of the tumor area), necrosis (necrosis if necrotic evidences including karyolysis, karyorhexis, and pyknosis are visible in more than 5% of the tumor area; no necrosis if necrotic evidences including karyolysis, karyorhexis, and pyknosis are visible in less than 5% of the tumor area), mitotic figures (cut-off value: 8 mitosis/10 HPF), and the portion of reserve cells were confirmed.

### 2.5. Immunohistochemical Analysis for Ki-67

The degree of immunostaining was assessed using a modified semi-quantitative assay as previously described [17]. The positively stained percentage of nuclei was scored as follows: 0: 0–5%, grade 0; I: 5–10%, grade 1; II: 11–25%, grade 2; III: 26–50%, grade 3; IV: >51%, grade 4.

### 2.6. Statistical Analysis

Statistical analysis was conducted using Prism 9 (GraphPad Software Inc., La Jolla, CA, USA) software. For comparison, Fisher’s exact test was used and presented as mean ± standard error (SE). The normality test was conducted by using a Shapiro–Wilk test and data were found to be non-normally distributed. Statistical significance was established using the Kruskal–Wallis test and data were presented as median ± interquartile range.

## 3. Results

This study included a total of 14 canine sebaceous adenomas and epitheliomas. Seven cases (50%) were identified as adenoma, four cases (29%) as borderline tumor, and three cases (21%) as epithelioma (Table 1). Adenoma cases showed an exophytic growth pattern in all cases. Borderline tumor cases displayed a lobulated growth pattern in two cases, trabecular in one case, and papillary growth pattern in one case. Epithelioma cases presented a lobulated growth pattern in all cases. 

### 3.1. Histological Comparison of Sebaceous Gland Tumors

**Figure 1:**
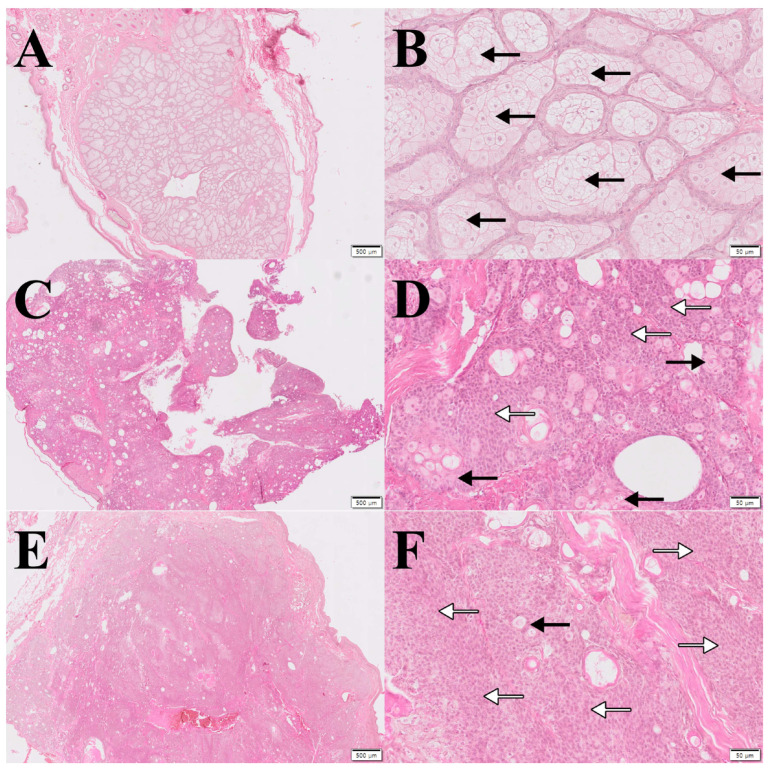
Representative histology of sebaceous adenoma, borderline tumor, and epithelioma. Sebaceous gland tumors were classified as (**A**,**B**) sebaceous adenoma, (**C**,**D**) borderline tumor, and (**E**,**F**) epithelioma based on the portion of sebocytes (black arrow) and reserve cells (white arrow). H&E stain; (**A**,**C**,**E**): ×10; (**B**,**D**,**F**): ×100.

The sebaceous adenoma was mainly composed of mature sebocytes surrounded by thin reserve cells (Figure 1A,B). In addition, the mature sebocytes and reserve cells mostly showed regular cells and nuclei. Although the borderline tumor was involved in adenoma, histological features were quite close to epithelioma (Figure 1C,D). The majority of the cells in the tumor were reserve cells and some sebaceous vacuolations demonstrate the origin of this tumor. There were more sebocytes than epithelioma, but the majority of them were immature. Sebaceous epithelioma was mainly composed of reserve cells with a basaloid appearance, some sebaceous vacuolations, and occasional cystic development (Figure 1E,F). Furthermore, the majority of the sebocytes were immature. 

**Figure 2:**
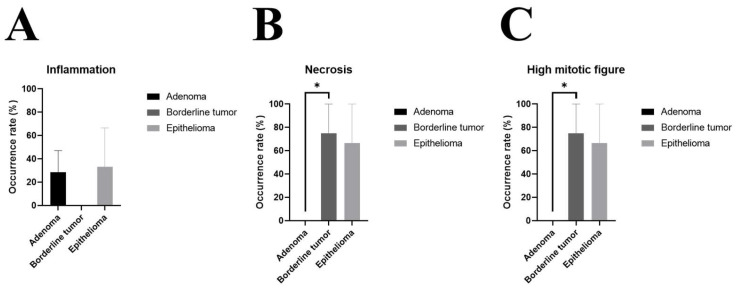
Comparison of histologic features of sebaceous adenoma, borderline tumor, and epithelioma. Sebaceous gland tumors were histologically diagnosed and the number of (**A**) inflammation, (**B**) necrosis, and (**C**) mitotic figures were evaluated. Data are shown as mean ± SE. *, *p* < 0.05. Table 2 summarizes the raw data to compare the histologic features of adenoma, borderline tumor, and epithelioma.

The occurrence rate of inflammation varied among adenoma (29%), borderline tumor (0%), and epithelioma (33%) (Figure 2A, Table 2A). However, necrosis was higher in borderline tumor (75%) than in adenoma (0%), and there was no difference between borderline tumor and epithelioma (67%) (Figure 2B, Table 2B). Furthermore, high mitotic figures were predominant in borderline tumor (75%) compared to adenoma (0%) but had no difference with epithelioma (67%) (Figure 2C, Table 2C).

### 3.2. Immunohistochemical Comparison of Sebaceous Gland Tumors

**Figure 3:**
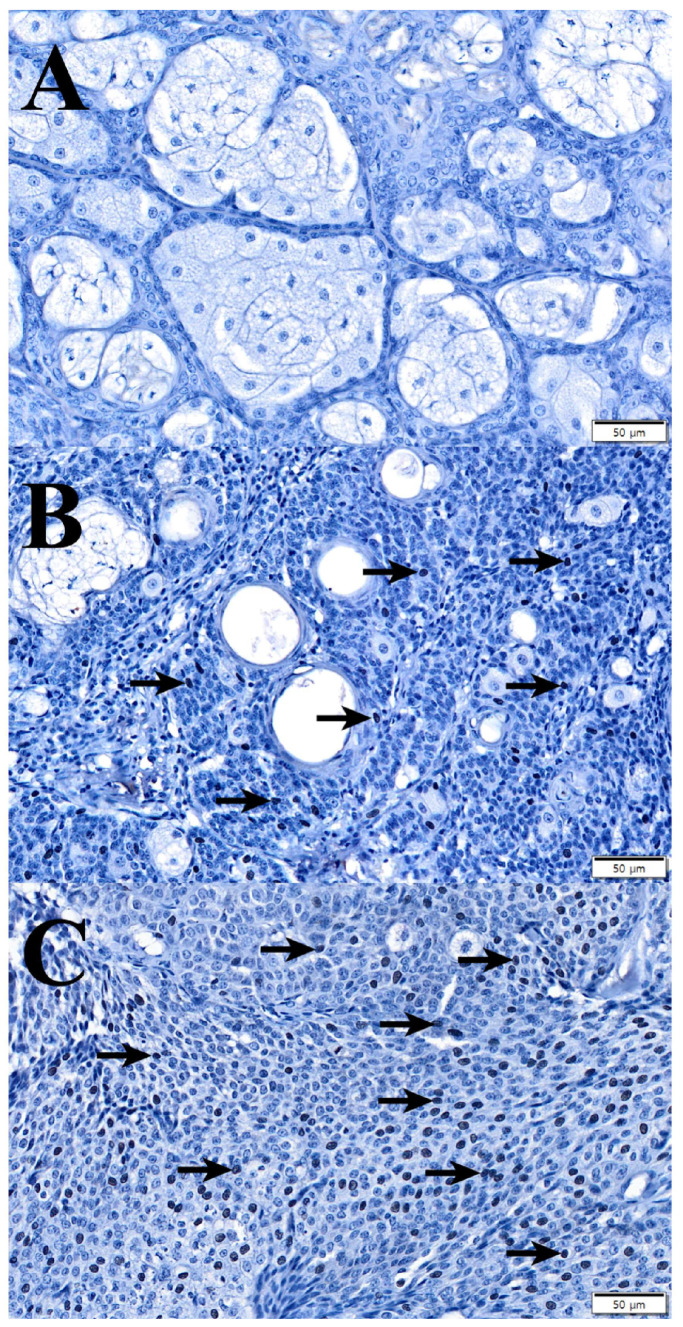
Representative IHC against Ki-67 in sebaceous adenoma, borderline tumor, and epithelioma. Sebaceous gland tumors were evaluated by considering the percentage of tumor cells positive for Ki-67 antigen (arrow) as mentioned in the materials and methods section. (**A**) Sebaceous adenoma; IHC score against Ki-67 0. (**B**) Borderline tumor; IHC score against Ki-67 III. (**C**) Sebaceous epithelioma; IHC score against Ki-67 IV. IHC against Ki-67 stain; ×100.

**Figure 4:**
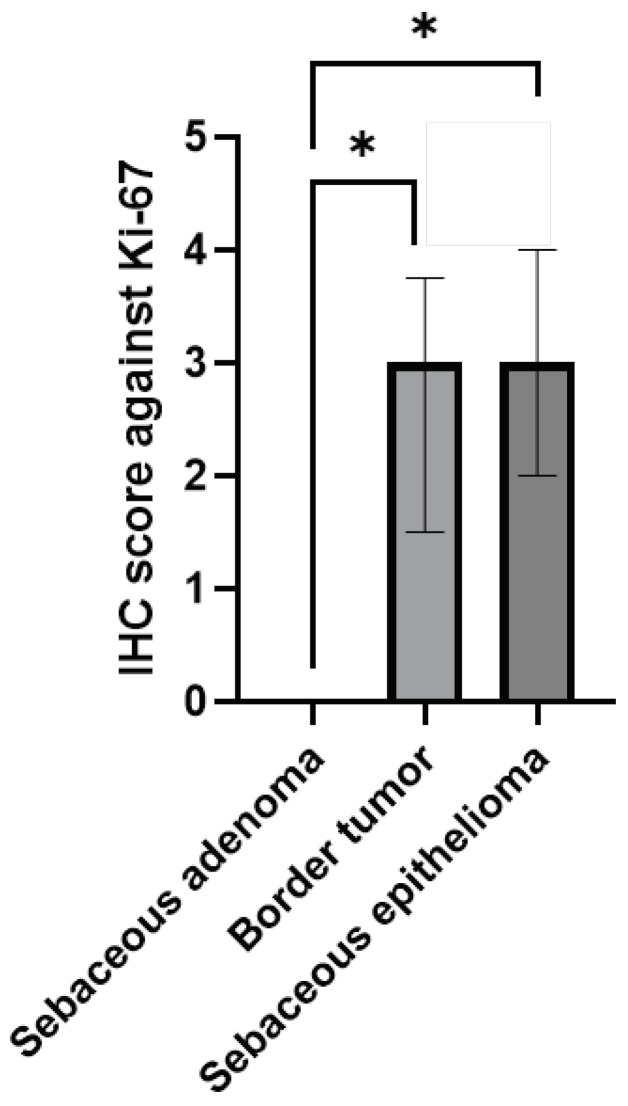
Comparison of Ki-67 immunolabelling between sebaceous gland tumors. Sebaceous gland tumors were histologically diagnosed and the IHC score against Ki-67 was semi-quantitatively graded. Data are shown as median ± interquartile range. *, *p* < 0.05.

Initially, we performed IHC against Ki-67 to measure the proliferation of tumors (Figure 3). Six cases of adenoma were grade 0 (86%) and one case was grade 2 (14%). One borderline tumor was grade 1 (50%), two were grade 3 (25%), and one was grade 4 (25%). Epithelioma had one case of grade 2 (33%), one case of grade 3 (33%), and one case of grade 4 (33%). There was a significant difference in the IHC score against Ki-67 between sebaceous adenoma and others, but there was no IHC score difference between borderline tumor and epithelioma (Figure 4). 

## 4. Discussion

Canine sebaceous adenoma and epithelioma are routinely treated surgically, and the prognosis is generally satisfactory. However, unlike adenoma, epithelioma is classified as a malignant tumor, so recurrence is possible even after surgical excision. According to a recent paper by Bettini et al., primary epithelioma metastases to the CNS and lung, implying that epithelioma is a potentially malignant tumor [1,10,18]. Therefore, histological misdiagnosis might lead to a discrepancy in prognosis; therefore, accurate diagnostic criteria are essential. Indeed, unlike adenoma, canine sebaceous epithelioma is identified when it has more than 90% reserve cells. Despite histological similarities with canine sebaceous epithelioma, sebaceoma is diagnosed in human medicine when it has more than 50% reserve cells, and this classification approach is known to be clinically beneficial [12]. Thus, we set tumors with 50 to 90% reserve cells as borderline tumors, and we aimed to clarify the diagnostic criteria by evaluating which characteristics the borderline tumor shares with adenoma and epithelioma.

Tumor-induced necrosis is more common in aggressive and faster-growing tumors due to the genetic instability of tumor cells [19]. It is well understood that the formation of hypoxic and ischemia circumstances due to aberrant vascularization results in energy deprivation, which leads to necrosis [19]. In particular, comedonecrosis, which arises in the center of the tumor, is a marker that indicates a poor prognosis in various malignancies, including breast carcinoma and prostate cancer [20,21]. Furthermore, tumor-induced necrosis is known as an indication of malignancy in human sebaceous tumors [22]. In our study, all necrosis was found to be comedonecrosis. Although there was no difference in inflammation among the tumors, necrosis was more common in borderline tumor and epithelioma than adenoma. This suggests that borderline tumor has characteristics similar to epithelioma and is potentially malignant.

Counting mitotic figures is the most well-known method for predicting tumor proliferation, and it is known that cancer-associated mutations increase the number of mitoses [23,24]. Therefore, the number of mitotic figures is frequently employed to assess the malignancy of various cancers such as mammary gland tumors and gastrointestinal stromal tumors [25,26]. In addition, the mitotic count of sebaceous adenoma was reported to be 11, while that of sebaceous carcinoma was reported to be 38 in 10 high-power fields, indicating that it is useful as an indicator of sebaceous tumor malignancy [27]. We found that borderline tumor tended to have higher mitotic figures than sebaceous adenoma, and there was no difference in mitotic figures between borderline tumor and sebaceous epithelioma. This also implies that borderline tumor has similar characteristics to epithelioma and has the potential to be malignant.

Ki-67 is a nuclear antigen found in cells of all differentiation phases (G1, S, and G2) and is utilized as a tumor diagnostic tool. Indeed, the Ki-67 index has been employed as a predictive biomarker in a variety of malignancies, including hepatocellular carcinoma, gastric cancer, and breast cancer [28,29,30]. In our experiment, since the IHC score against Ki-67 was much higher in borderline tumor than in adenoma and there was no difference between borderline tumor and epithelioma, we confirmed that borderline tumor and epithelioma have comparable molecular characteristics. Comparable to our findings, Bongiovanni et al. demonstrated 2.1% Ki-67 positive cells in sebaceous adenoma compared to 12.43% in malignant epithelioma [31]. In contrast, Hohsteter et al. reported no significant difference in the Ki-67 index between sebaceous adenoma (16.67%) and epithelioma (18.6%) [32]. Our data suggest that, due to the high IHC score against Ki-67 in borderline tumor, the conventional criteria for diagnosing sebaceous adenoma from epithelioma are not appropriate.

Histologically and immunohistochemically, human sebaceoma and canine sebaceous epithelioma have several similarities and differences. Histologically, human sebaceoma and canine sebaceous epithelioma share a predominance of basaloid cells over mature sebocytes. In addition, immunohistochemically, human sebaceoma is positive for several cytokeratins, including CK5 and CK14, and shows relatively low positivity for these cytokeratins in sebaceous carcinoma [33]. CKAE1/AE3, CK5, and CK14 are often positive in basal and basaloid cells and their reduction is seen at earlier stages of malignant progression [34]. This is very similar to canine sebaceous epithelioma, which is also positive for CKAE1/AE3, CK14, and CK5/6. However, human sebaceoma has been shown to have a lower Ki-67 than sebaceous carcinoma, whereas canine sebaceous epithelioma has been shown to be similar to sebaceous carcinoma [31,35]. Unfortunately, there is insufficient research on canine sebaceous epithelioma to make further comparisons between human sebaceous epithelioma and canine sebaceous epithelioma. Histologically, sebaceous carcinoma in humans is divided into lobulated, papillary, and trabecular growth patterns, and sebaceoma is divided into carcinoid-like, rippled, reticulated, cribriform, and Verocay body-like patterns, but there is no clear classification in canine sebaceous tumors [36,37,38,39]. In addition, human sebaceoma has low levels of p53, which are elevated in sebaceous carcinoma, and high levels of Bcl-2 and p21, which are associated with a worse prognosis. Principal component analysis confirms that sebaceous hyperplasia, sebaceous adenoma, and sebaceoma have closely related features, but this has not been studied in canine sebaceous tumors [35]. 

However, our study has a limitation in that it only included 14 tumor specimens. In addition, even though the tumor has a hormonal predisposition, analyses of the sex and neutralization status were not conducted due to the difficulty in data collection. Further studies are needed to clearly establish the histological and immunohistochemical characteristics of sebaceous adenoma and epithelioma, as well as the sex differences in tumor incidence. Moreover, additional studies will help us to understand the pathophysiology of sebaceous tumors in both humans and dogs and will be of great benefit to translational medicine.

## 5. Conclusions

In conclusion, we established a more reliable diagnostic criterion of a 50% cut-off value of reserve cells for canine sebaceous epithelioma than the previous system. Unlike sebaceoma, which is a benign tumor in humans, epithelioma is a malignant tumor that requires a more precise diagnosis in the canine [12,40]. An improved diagnostic and prognosis standard can be developed with a more accurate diagnostic criterion construction by confirming it with several biomarkers from a larger number of patients. We hope that this will aid in adequate treatment and a favorable prognosis.

## Figures and Tables

**Table 1:** Histological classification and profiling of 14 canine sebaceous gland tumors.

Case	Breed	Age	Site	Growth Pattern	Reserve Cell Portion	Diagnosis
#1	York Terr	12Y	Skin	Exophytic	<10%	Adenoma
#2	Shih-Tzu	9Y8M	Lt. Front limb	Exophytic	<10%	Adenoma
#3	Maltese	10Y	Hind limb	Exophytic	<10%	Adenoma
#4	Maltese	7Y9M	Between eyebrows	Exophytic	<10%	Adenoma
#5	Poodle	8Y	Nose bridge	Exophytic	<10%	Adenoma
#6	Maltese	16Y2M	Hip subcutis	Exophytic	<10%	Adenoma
#7	Bichon Frise	8Y	Tragus (ear)	Exophytic	<10%	Adenoma
#8	Cocker Spaniel	14Y	Ear	Lobulated	50–60%	Border tumor
#9	Cocker Spaniel	12Y	Rt. Ear	Trabecular	60–70%	Border tumor
#10	Mixed	13Y	Rt. Knee	Lobulated	60–70%	Border tumor
#11	-	7Y8M	Tail	Papillary	80–90%	Border tumor
#12	Cocker Spaniel	11Y	Rt. Shin	Lobulated	90–95%	Epithelioma
#13	Shih-Tzu	10Y	Rt. Forelimb	Lobulated	90–95%	Epithelioma
#14	Shih-Tzu	10Y	Mandible	Lobulated	>95%	Epithelioma

Note: Y, year; M, month; Lt, left; Rt, right.

**Table 2:** Comparison of (A) inflammation, (B) necrosis, and (C) mitotic figures between sebaceous adenoma, borderline tumor, and epithelioma.

**A**			
	Inflammation	No inflammation	*p*-value
Adenoma	2	5	0.4909
Borderline tumor	0	4	
Adenoma	2	5	>0.9999
Epithelioma	1	2	
Borderline tumor	0	4	0.4286
Epithelioma	1	2	
**B**			
	Necrosis	No necrosis	*p*-value
Adenoma	0	7	0.0242
Borderline tumor	3	1	
Adenoma	0	7	0.0667
Epithelioma	2	1	
Borderline tumor	3	1	>0.9999
Epithelioma	2	1	
**C**			
	High mitotic figure	Low mitotic figure	*p*-value
Adenoma	0	7	0.0242
Borderline tumor	3	1	
Adenoma	0	7	0.0667
Epithelioma	2	1	
Borderline tumor	3	1	>0.9999
Epithelioma	2	1	

## Data Availability

The data presented in this study are available on request from the corresponding author. The data are not publicly available to preserve the privacy of the data.
